# Supplementary material for: Patterns of Intron Gain and Loss in Fungi
Source: PLoS Biol. 2004 Nov 30;2(12):e422. doi: 10.1371/journal.pbio.0020422 (PMC532390; doi:10.1371/journal.pbio.0020422)
Supplement: Table S1 — Also available at http://genes.mit.edu/NielsenEtAl/. (4.3 MB ZIP). [file pbio.0020422.st001.zip › NielsenEtAl/html/998.html]

AN3658.1.NCU09811.1.MG05739.1.FG09376.1


```
 CLUSTAL W (1.82) Multiple Sequence Alignments - Introns Inserted


Sequence 1: AN3658.1	485 aa
Sequence 2: FG09376.1	504 aa
Sequence 3: NCU09811.1	484 aa
Sequence 4: MG05739.1	625 aa
Alignment Length: 654 aa
Number Identitical Residues: 116 aa
Alignment Score (without introns) 7620


MG05739.1 	---MTDPS-VPAP-------AVSADATKDETTAVQPQHSR------PPSPEVVEEKE---
NCU09811.1	MTLSPDPSTAPAPSRQNASFPPSNQAPMANARAAAEPESKSLFDESPEDSDAVKEDE---
FG09376.1 	---MADLEEKKTSQSPENDVGQTRRSQDTTTTEEGVITQVQQQDQQISTSEKDNGDMFPP
AN3658.1  	---MADHRSLDIIRSHNDGSGQDIQE----SSEEKYPENID--DERTSTVAAKSGDL---
          	    .*          . .           :       . .  ..        . .    

MG05739.1 	-------FKEGGYGW2VIVLAVHLLNAHTWGINA~SYSVFLAYYLRSGAFAGASPIAYAF
NCU09811.1	-------FKEGGYGW2VVVFGVWLVNMHTWGLIS0SYAIFLAYYLRSGSIIGASPLFFAF
FG09376.1 	QNDVPPPPPNGGYGW~VCTACVAIINAHTWGLNS~SYGVFLAHYIQNDTFPGATSLHYAF
AN3658.1  	-------PPDGGYGW0------------------~SYGVFLAYYLSHDVFPNTSALMYAF
          	         :*****                    **.:***:*:  . : .::.: :**

MG05739.1 	VGGLSISIA1LGISPIATISVREFGTRNTLLLGSAIIT2IAFIGASFTTQMWQLVLSQGI
NCU09811.1	IGGLSTSMA1LLVSPLATYSIKTLGTRITLFIGAVFEA2GSFIGASFSTHAWQLLLSQGV
FG09376.1 	VGSLSIACA~MLVSPLATMCVRDFGTKPTLFFGIILET~ASLICASFASKIYQLFLTQGV
AN3658.1  	TGGLSISCA~LLVAPLATYLIHVLGTRAVLNIGVFFET~LSLIASSFVSQRWQIFLSQGV
          	 *.** : * : ::*:**  :: :**: .* :*  : :  ::* :** :: :*:.*:**:

MG05739.1 	AFGLGMGFTFVASVGVVPQWFVKRRSLANGLGAGGSGMGGLIYSLATNAMIKDLGLSWTF
NCU09811.1	CFGLGMGFCFTATVGIVPQWFTKRRSFANALATGGSGFGGLTYALGANAMIANLGLEWAF
FG09376.1 	LFGLGMGFLFVPSVGIVPQWFTTRRSLANGISAAGSGLGGLLYSLAAGAIIRNISLQWAY
AN3658.1  	CFGIGMGFLFVGSVGITPQWFHRRRSLAMGINAAGSGLGGLTYSLAVGAIIPRLGLSWAF
          	 **:**** *. :**:.****  ***:* .: :.***:*** *:*...*:*  :.*.*::

MG05739.1 	RVLALIVLVVNCISSVLVKDRNKELGTVQVAFHKDLFKRPEYYLLIAWAFFSILGYTITV
NCU09811.1	RILAITCFTVNGCVSLIIKDRNKAVGAKHIAFHKDLFFQPEYWLFVGWGFFGIIGYIIVV
FG09376.1 	RILGIIVFVVNIICTILVKDRNKIVGSRQAPFDLSLFKRPEFHLLLGYGWFSMLGYVVLI
AN3658.1  	RILGIVSCVVNLVCANLLRDRNTAVGSRFKAFHFPLFKRPEFLLFLGWGIFSMLGYVAIL
          	*:*.:   .**   : :::***. :*:   .*.  ** :**: *::.:. *.::**   :

MG05739.1 	FSLSDYCQTVGFTATQGSVVAALYSM~SQGIGRPIIGHFSDSWGRINVAAIGTLISAVAS
NCU09811.1	FSIADFALQAGFTSNQASLASAMFNL1SQALGRPAIGLLSDRLGKMNVAGIGTLIASVVA
FG09376.1 	FSLANYANFIGLDASQAAMVSAIFNL~GQAVGRPFIGYFSDRTGRINMACATTWLAAILV
AN3658.1  	FSMSNFAVSVGLSAHQASIISALLNL~GQGLGRPFVGMFSDKLGRINIATFLSFLCGVFC
          	**::::.   *: : *.:: :*: .: .*.:*** :* :**  *::*:*   : :..:  

MG05739.1 	FTLWIFAGPHFGGVIVFALLGVFTGILWATIGPVSAEVVGLELLPSA~LSVIWLVLAVPA
NCU09811.1	FFLWIFAGKYFAGLIIYALFGAVAGIIWPCVGPVGAEVVGIQLLPAA1LSLYWIGLVFPA
FG09376.1 	FAIWINAKSYGLLLFFTIVGGAMAGTYWATVGPVTAEVVGLADVPAA~LNLTWLVISLPC
AN3658.1  	LLIWTFARSMGLVSFFAVLVGTVAGTYWATVAPVLAEIIDLKDLPGG~LSITWIVLVAPT
          	: :*  *       :.  : *..:*  *. :.** **::.:  :*.. *.: *: :  * 

MG05739.1 	TF~AMVIGVSLR--VPGPGGYLFVQVFVGLMYLFAFTSA1WFLRGWKLWQLENLDRNKSR
NCU09811.1	TF2AEVIGLSLKREMIGAGVYLNVQVFTGFMFFASFLSM1WLLRSWKLRQMEYLGLYEKG
FG09376.1 	TF~STPIALQIVAG---TGKYIGAQIFVGFMYIAGGLCL~FFLRGWKIGELAEIAR----
AN3658.1  	TV~AEPIALLLRDNDSPSRIYLHVQLFTGLMYIAAAVCL~WTVRGWKVGENLRVKQEKSA
          	*. :  *.: :      .  *: .*:*.*:*:: .  .  : :*.**: :   :   .. 

MG05739.1 	GAGVMRNDDAAVTGDTE1TSTPNARAGEEMLFTHLHDSRHL1PPIRQQRNH~RLQSPAAR
NCU09811.1	QEAAIHN--VAVIHPSE~ES---------------------~------KHE0RL------
FG09376.1 	-ATNHDPEEISRVRSQA~SGAISIK----------------~------SRR~IGRK----
AN3658.1  	VAVETDKKDESVVLQQQ~SGGTGAS----------------~------RSQ~QSTKTAVW
          	       .. :        .  .                           .    ..:  

MG05739.1 	LLTSFIGIYTKPPRKAHKPHQNVVVLASDRWQQRLGHIRA1LRQRPA0GQAVDEVEYSRT
NCU09811.1	----------------------------------------~------~------------
FG09376.1 	---RMLGECYKPGKV-------------------------~------~------------
AN3658.1  	YPHALMRGMVSAKKV-------------------------~------~------------
          	          ..                                                

MG05739.1 	SRPGGVEGTSQEAWRGSEVHGVGVQGVGGLLVANKEEAEGAKVSGYCGAPDWVGEAIGPL
NCU09811.1	------------------------------------------------------------
FG09376.1 	------------------------------------------------------------
AN3658.1  	------------------------------------------------------------
          	                                                            

MG05739.1 	RSGTIGI
NCU09811.1	-------
FG09376.1 	-------
AN3658.1  	-------
          	
```
